# Clinical-grade generation of peptide-stimulated CMV/EBV-specific T cells from G-CSF mobilized stem cell grafts

**DOI:** 10.1186/s12967-018-1498-3

**Published:** 2018-05-09

**Authors:** Regina Gary, Michael Aigner, Stephanie Moi, Stefanie Schaffer, Anja Gottmann, Stefanie Maas, Robert Zimmermann, Jürgen Zingsem, Julian Strobel, Andreas Mackensen, Josef Mautner, Andreas Moosmann, Armin Gerbitz

**Affiliations:** 1 Dept. of Hematology/Oncology, University Hospital of Erlangen, Ulmenweg 18, 91054 Erlangen, Germany; 2 DZIF Research Group Host Control of Viral Latency and Reactivation (HOCOVLAR), Helmholtz Zentrum München, Marchioninistr. 25, 81377 Munich, Germany; 3 Center for Clinical Studies CCS, University Hospital of Erlangen, Krankenhausstr. 12, 91054 Erlangen, Germany; 4 Department of Transfusion Medicine and Hemostaseology, University Hospital of Erlangen, Krankenhausstr. 12, 91054 Erlangen, Germany; 5 Clinical Cooperation Group Pediatric Tumor Immunology, Helmholtz Zentrum München, and Technical University of Munich, Marchioninistr. 25, 81377 Munich, Germany; 6 Department of Hematology, Oncology and Tumorimmunology, Charité Berlin, Berlin, Germany

**Keywords:** Stem cell transplantation, Allogeneic, CMV, EBV, Reactivation, T cell, Adoptive transfer

## Abstract

**Background:**

A major complication after allogeneic hematopoietic stem cell transplantation (aSCT) is the reactivation of herpesviruses such as cytomegalovirus (CMV) and Epstein–Barr virus (EBV). Both viruses cause significant mortality and compromise quality of life after aSCT. Preventive transfer of virus-specific T cells can suppress reactivation by re-establishing functional antiviral immune responses in immunocompromised hosts.

**Methods:**

We have developed a good manufacturing practice protocol to generate CMV/EBV-peptide-stimulated T cells from leukapheresis products of G-CSF mobilized and non-mobilized donors. Our procedure selectively expands virus-specific CD8+ und CD4+ T cells over 9 days using a generic pool of 34 CMV and EBV peptides that represent well-defined dominant T-cell epitopes with various HLA restrictions. For HLA class I, this set of peptides covers at least 80% of the European population.

**Results:**

CMV/EBV-specific T cells were successfully expanded from leukapheresis material of both G-CSF mobilized and non-mobilized donors. The protocol allows administration shortly after stem cell transplantation (d30+), storage over liquid nitrogen for iterated applications, and protection of the stem cell donor by avoiding a second leukapheresis.

**Conclusion:**

Our protocol allows for rapid and cost-efficient production of T cells for early transfusion after aSCT as a preventive approach. It is currently evaluated in a phase I/IIa clinical trial.

**Electronic supplementary material:**

The online version of this article (10.1186/s12967-018-1498-3) contains supplementary material, which is available to authorized users.

## Background

Reactivation of cytomegalovirus (CMV) and Epstein–Barr virus (EBV) worsens outcomes of allogeneic stem cell transplantation (aSCT) and remains a major obstacle to its success [1]. Within the first 100 days after aSCT, 40–50% of patients reactivate CMV, and up to 40% of all patients reactivate EBV after aSCT as determined by virus-specific PCR of cells of the peripheral blood (PB). Approximately 95% of donors and patients are seropositive for EBV, and 40–70% for CMV [2]. Both CMV and EBV reactivation after aSCT are associated with increased mortality. Reactivation of EBV bears the risk of EBV-associated post-transplantation lymphoproliferative disease [3]. Reactivation of CMV can cause pneumonia with high mortality. Therefore both viruses require preemptive treatment upon reactivation in patients after aSCT [4].

Specific antiviral therapy is only available for the treatment of CMV. However, all drugs available (Ganciclovir, Foscarnet, Cidofovir, and others) display strong side effects including bone marrow and kidney failure. Furthermore, they frequently require inpatient treatment thereby compromising quality of life and most importantly do not solve the underlying problem of missing immunological control. For EBV, no approved specific therapeutic option exists. Off-label use of Rituximab, a B-cell depleting antibody, is increasing and seems to be effective [5–7]. However, Rituximab induces long lasting B-cell depletion resulting in frequent and obligatory transfusion of immunoglobulins. Similarly to the treatment of CMV, the fundamental problem of the lack of immunological control is not addressed with this therapy. As all antiviral therapies fail to boost the immune system, relapse of reactivation is frequent and repeated treatments are required, strongly contributing to the high costs of aSCT.

The rationale of strengthening specific T-cell immunity for both prevention and therapy of CMV and EBV reactivation therefore represents an intriguing therapeutic option. Several groups have shown that CMV- or EBV-specific T cells can be isolated or enriched from seropositive donors, and mediate viral control in aSCT patients after adoptive transfer [8–14]. Depending on the method of isolation, virus-specific T cells are only available in a minority of donor-patient pairs, their specificity is limited to single viral antigens or epitopes, or their preparation may be inconveniently long and laborious. Here, we describe a clinical grade protocol for manufacturing multi-epitope CMV/EBV-specific T cells suitable for application after aSCT. We use a generic set of peptides representing dominant CMV and EBV CD8+ and CD4+ T-cell epitopes from different viral antigens of each virus, presented by different HLA allotypes. Thus, this protocol is applicable in more than 80% of European donors, and has a high likelihood to enrich their dominant virus-specific T-cell populations. We applied this procedure to G-CSF mobilized stem cell grafts and non-mobilized apheresis products and show that it is equally effective in the relative expansion of CMV/EBV-specific T cells. As a result, CMV/EBV-specific T cells are available shortly after transplantation within 14 days (plus the time required for microbial safety monitoring) if G-CSF mobilized stem cells are used as a T-cell source, avoiding a second apheresis of the donor. The protocol is easily applicable within clean room facilities and can be modified according to preferences of the manufacturer. This manufacturing protocol is currently used in an ongoing phase I/IIa clinical trial for prevention of CMV/EBV-reactivation after aSCT (EudraCT 2012-004240-30).

## Methods

### Donor selection

Donor selection was based on a positive CMV and EBV serostatus and on at least one matching HLA class I allele for both peptide pools.

### Cell culture

Collection of PBMC from G-CSF mobilized stem cell grafts were approved by the local ethics committee (Ref. No 4388). Only remaining cells from the tubing set and transfusion bag after transfusion of the stem cell graft were used. After transfusion of the stem cell graft, the line was disconnected from the patient and connection was sealed with a CombiStop tip. The transfusion bag and tubing set were flushed twice with 50 ml 0.9% NaCl solution through a three-way connection of the transfusion line. Leukapheresis products were obtained from non-mobilized volunteer donors and a small fraction was processed. On average, 1.3 × 10^9^ ± 0.4 × 10^9^ (Mean ± SD) CD45+ cells were cryopreserved as starting material for cell culture. For the clinical trial, autologous serum is obtained from the donors before apheresis using EVA bags (Maco Pharma International GmbH, Langen, Germany) for blood collection. For establishment, serum was collected by serum tubes with clotting activator (Sarstedt AG & Co GK, Nümbrecht, Germany). Serum was separated from clotted blood by centrifugation (three times 1800*g*, 10 min). PBMC from leukapheresis products were purified through Ficoll (GE Healthcare Europe GmbH, Freiburg, Germany) gradient centrifugation (800*g*, 20 °C, 20 min). Cells were washed twice with PBS, counted and cryopreserved at a concentration of 100 × 10^6^/ml in a 20% HSA solution containing 10% DMSO.

**Fig. 1:**
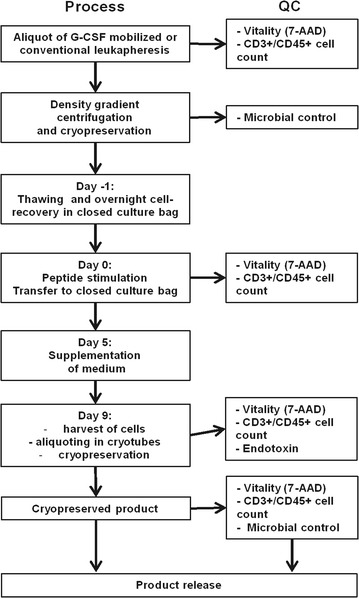
Overview of the manufacturing process and quality controls

For T-cell stimulation, cryopreserved cells were thawed and recovered overnight in serum-free CellGro DC Medium (CellGenix GmbH, Freiburg, Germany) at a concentration of 1–4 × 10^6^/ml. After 18–24 h cells were harvested, counted and stimulated in two separate fractions with CMV and EBV peptide pools at a concentration of 1 µg/ml per peptide in standard Falcon tubes in 20 ml of prewarmed (37 °C) CellGro DC medium. After 2 h of incubation at 37 °C, remaining peptides were washed out. Peptides were purchased from JPT Peptides Technologies GmbH (Berlin, Germany) at a purity of ≥ 95% (see Table 3). Both cell fractions were united, washed with medium again, and transferred into a culture bag (MACS GMP Cell Differentiation Bag 500 ml, Miltenyi Biotec GmbH, Bergisch Gladbach, Germany) at a concentration of 2.5 × 10^6^ cells/ml. Cells were cultured for 9 days at 37 °C in CellGro DC Medium with 50 IU/ml IL-2 (Aldesleukin, Novartis Pharma GmbH, Nürnberg, Germany), 1% (v/v) GlutaMAX™ (Gibco by Life Technologies GmbH, Darmstadt, Germany) and 1% (v/v) autologous serum (“complete medium”). On day 5 after peptide stimulation, complete medium (volume on day 0 multiplied by 1.5) was supplemented. On day 9, cells were harvested from the culture bag, analyzed and divided in appropriate doses. Cells were stored in the gas phase over liquid nitrogen until usage. The workflow is depicted in Fig. 1.

### Flow cytometric analysis

All antibodies were purchased from BD Biosciences (Heidelberg, Germany) unless otherwise specified.

For phenotypic analysis, cells were stained with anti-CD8 FITC (clone SK1), anti-CD25 PE (clone 2A3), anti-CD14 PerCP (clone MφP9), anti-CD56 APC (clone B159), anti-CD19 PE-Cy7 (clone SJ25C1), anti-CD4 APC-Cy7 (clone RPA-T4), anti-CD3 V450 (clone UCHT1), and anti-CD45 V500 (clone HI30). Gating included the exclusion of debris in a SSC vs FSC plot. Leukocytes were gated in a CD45 vs SSC dot plot as CD45+ cells. The CD45^high^/SSC^low^ population was termed lymphocytes, and was clearly distinguished from monocytes and granulocytes. Within CD45^high^/SSC^low^ cells, subsets were characterized by expression of CD3 (T cells), CD19 (B cells), and CD56 (negative for CD3) (NK cells). Monocytes were gated as CD14+ SSC^intermediate^ cells. Cell number in whole blood was determined by FACS using fluorescent beads (Trucount™ Tubes, BD Biosciences, Heidelberg, Germany).

For analysis of CMV- and EBV-specific T cells, 1 × 10^6^ PBMC were stained with peptide-loaded multimers (ProImmune Ltd., Oxford, United Kingdom) as indicated according to the manufacturer’s recommendation. Cells were stained in addition with anti-CCR7 FITC (clone 150503, R&D Systems), Fluorotag PE, anti-CD8 PerCP (clone SK1), anti-CD62L APC (clone DREG-56), anti-CD45RA PE-Cy7 (clone HI100), anti-CD4 APC-Cy7 (clone RPA-T4), and anti-CD3 V450 (clone UCHT1). Lymphocytes were gated in the SSC vs FSC dot plot. T cells were defined as CD3+/SSC^low^ and further subdivided into CD4+ and CD8+ subsets. Pentamer-binding cells were determined as proportion of CD8+ T cells. Cells were analyzed subsequently after staining using a FACS Canto II (Becton–Dickinson, Heidelberg, Germany).

### ELISpot assay

For ELISpot assays, cryopreserved T cells (day 9 of culture) and PBMC (day 0 of culture) were thawed for overnight recovery in RPMI1640 medium containing 10% human AB serum. At the same day, a Multiscreen plate (mixed cellulose ester membrane, MAHAS4510, Merck Millipore, Merck KGaA, Darmstadt, Germany) was coated with anti-IFN-γ antibody (clone NIB42, BD) in PBS (15 µg/ml, 50 µl/well). The plate was incubated for 12–18 h at 4 °C, then the antibody solution was removed, the plate was filled with medium containing 10% human AB serum, and kept for 2 h at 37 °C for blocking. PBMC served as APC and as background control. After blocking, PBMC alone, or PBMC and expanded T cells were plated in different T-cell concentrations and stimulated with CMV peptide pool, EBV peptide pool, or human gp100 control peptide at a concentration of 0.5 µg/ml. Plates were incubated for 19–24 h at 37 °C. After incubation, plates were washed six times with PBS + 0.05% Tween 20 (Sigma-Aldrich Chemie GmbH, Munich, Germany). Then, anti-IFN-γ-biotin antibody (clone 4S.B3, BD) in PBS (50 µl/well, c = 0.3 µg/ml) was added and plates were incubated overnight at 4 °C. Plates were washed four times with PBS, and streptavidin alkaline phosphatase (Bio-Rad Laboratories GmbH, Munich, Germany) was added in a dilution of 1:1600 in PBS. Plates were incubated for 2 h at room temperature and washed with PBS. Colorimetric AP substrate was added and incubated at room temperature in the dark for 10–30 min, depending on assay-specific spot development. Spots were quantified by a Bioreader 3000 System (Bio-Rad).

### Cell counting

For microscopic cell counting, trypan blue-stained cells were counted in a Neubauer chamber. Cells were diluted in PBS depending on cell concentration to obtain a concentration of 0.5–1.0 × 10^6^ cells/ml. Trypan blue was used in a concentration of 0.4%.

### Storage in the gas phase over liquid nitrogen

Cells were cryopreserved in pre-cooled cryopreservation medium of 90% HSA-solution (Serum Albumin 20% Behring, CSL Behring GmbH, Hattersheim am Main, Germany) and 10% DMSO (100%, pharmacy of the University Hospital of Erlangen, Germany).

### Analysis of data

Data were analyzed and graphically represented using GraphPad Prism V and Microsoft Excel 2010. Flow cytometry data were analyzed either by FACS Diva (BD), Kaluza (Beckman Coulter), or FlowJo (FlowJo LLC, Oregon, USA). For statistical analysis, the Mann–Whitney U test was used.

### Sterility testing

Microbial contamination of products of mobilized donors was monitored by aerobic and anaerobic culture (BacTec sytem), and by eubacterial PCR. For the clinical trial, microbial control consists of monitoring of microbial growth according to Ph. Eur. 2.6.27, mycoplasma PCR according to Ph. Eur. 2.6.7, and endotoxin detection according to Ph. Eur. 2.6.14, performed by a subcontracted laboratory (Eurofins BioPharma Product Testing Munich GmbH, Planegg/Munich, Germany).

## Results

We established a GMP-compliant process for manufacturing of CMV/EBV-specific T cells by peptide stimulation of leukapheresis products. The workflow is outlined in Fig. 1. Details of manufacturing are provided in Materials and Methods. We investigated this process with a particular focus on stimulation of T cells from leukapheresis products from G-CSF mobilized donors to ensure availability of the product as early as possible after transplant and, for donor safety, to avoid a second apheresis procedure.

### Cellular composition of G-CSF mobilized and non-mobilized peripheral blood preparations

**Fig. 2:**
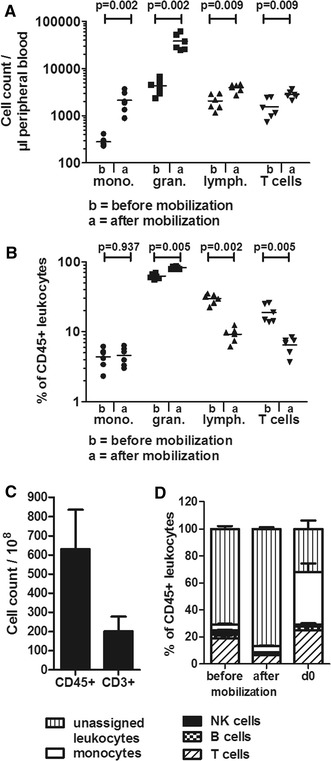
Influence of G-CSF mobilization on cell composition. Peripheral blood of donors before and after mobilization, and leukapheresis products from G-CSF mobilized donors after Ficoll and cryopreservation were analyzed. **A** Flow cytometry was used to determine absolute cell counts of monocytes (mono.), granulocytes (gran.), lymphocytes (lymph.), and T cells per µl of peripheral blood before (“b”) and after (“a”) G-CSF mobilization in stem-cell donors, n = 6. **B** Relative subset composition of leukocytes in donors before and after G-CSF mobilization (n = 6). **C** Absolute cell count of CD45+ and CD3+ cells in stem cell grafts. The mean value ± SD of 15 preparations from 15 donors is shown. **D** Cell composition of leukocytes before and after mobilization in peripheral blood and after cryopreservation at day 0 before peptide stimulation. The proportion of T cells, B cells, NK cells, monocytes, and remaining unassigned leukocytes is shown at the three different time points, n = 5. **A**, **B** Statistical analysis was performed using the Mann–Whitney U test

Standardized T-cell expansion protocols usually rely on leukapheresis products from untreated healthy volunteer donors. To be able to generate donor-derived virus-specific T cells for preventative use early after aSCT, we investigated the expansion of CMV- and EBV-specific T cells from aliquots of G-CSF mobilized hematopoietic stem cell grafts as a T-cell source. As shown in Fig. 2A and Table 1, analysis of absolute cell numbers in the peripheral blood before (b) and after (a) G-CSF mobilization revealed a strong increase of monocytes and granulocytes whereas absolute numbers of lymphocytes were moderately increased. Accordingly, the percentage of lymphocytes among leukocytes in the peripheral blood decreased after administration of G-CSF due to the massive increase in granulocytes (Fig. 2B, Table 1). Likewise, absolute numbers of T cells increased whereas their proportion decreased. In line with our findings, Rodriguez-Cortes et al. describe an extensive increase of leukocytes after G-CSF mobilization, including a moderate increase of lymphocytes [15]. Our observations are also consistent with data from Chevallier et al. [16] and Sica et al. [17].

**Table 1:** Cellular composition of peripheral blood before and after mobilization

	Granulocytes	Monocytes	Lymphocytes	T cells	B cells	NK cells
Cell count (n = 6)						
Before mobilization	43.3 ± 16.2 × 10^2^/µl	2.8 ± 0.7 × 10^2^/µl	20.6 ± 7.6 × 10^2^/µl	15.4 ± 7.5 × 10^2^/µl	2.2 ± 0.7 × 10^2^/µl	2.3 ± 0.4 × 10^2^/µl
After mobilization	383.9 ± 153.9 × 10^2^/µl	21.4 ± 10.6 × 10^2^/µl	39.7 ± 7.4 × 10^2^/µl	28.3 ± 5.4 × 10^2^/µl	5.2 ± 2.0 × 10^2^/µl	3.9 ± 2.1 × 10^2^/µl
Percentage (n = 6)						
Before mobilization (%)	62.3 ± 5.5	4.4 ± 1.4	29.8 ± 4.8	18.8 ± 5.5	2.8 ± 0.5	3.1 ± 1.2
After mobilization (%)	82.4 ± 3.9	4.6 ± 1.4	9.2 ± 2.2	6.5 ± 1.8	1.1 ± 0.5	0.8 ± 0.2

Whole peripheral blood of donors before and after G-CSF mobilization was analyzed by flow cytometry and Trucount™ tubes

To obtain the desired number of 3.0–5.0 × 10^6^ T cells per dose (5.0 × 10^4^/kg body weight) and a sufficient number of cells for quality control after manufacturing, 1.3 × 10^9^ ± 0.4 × 10^9^ (mean ± SD) CD45+ cells were cryopreserved for the use as starting material for the T-cell stimulation process, both for mobilized and non-mobilized donors. On average, grafts from unrelated or related G-CSF mobilized donors in our institution contain 63.0 × 10^9^ ± 20.7 × 10^9^ CD45+ cells and 20.1 × 10^9^ ± 7.8 × 10^9^ CD3+ T cells (n = 15, Fig. 2C). Therefore, on average, 3.2% of the graft would be required to obtain 2 × 10^9^ CD45+ cells as starting material. Our manufacturing process involves cryopreservation of the apheresis product after Ficoll density gradient centrifugation. This step was introduced to generate flexibility in manufacturing especially with regard to clean room availability but also includes the advantage of an additional strong reduction of the number of granulocytes, since these may have a negative impact on later T-cell expansion. When the relative cellular composition of peripheral blood cells before and after G-CSF mobilization was compared to the apheresis product after cryopreservation, thawing and overnight recovery (d0), the fraction of granulocytes (and their immature precursors) was strongly reduced in the day 0 product, monocytes were increased, and the proportions of T and B cells became comparable to peripheral blood before G-CSF mobilization. Cellular composition on day 0 displayed a preponderance of monocytes and T cells. The percentage of B cells and NK cells declined in the PB after mobilization (Fig. 2D). Cell recovery after thawing and recovery was 227 × 10^6 ^± 160 × 10^6^ cells for mobilized donors, 690 × 10^6 ^ ± 221 × 10^6^ for non-mobilized donors (Mean ± SD).

### T-cell expansion after peptide stimulation and cultivation for 9 days

**Fig. 3:**
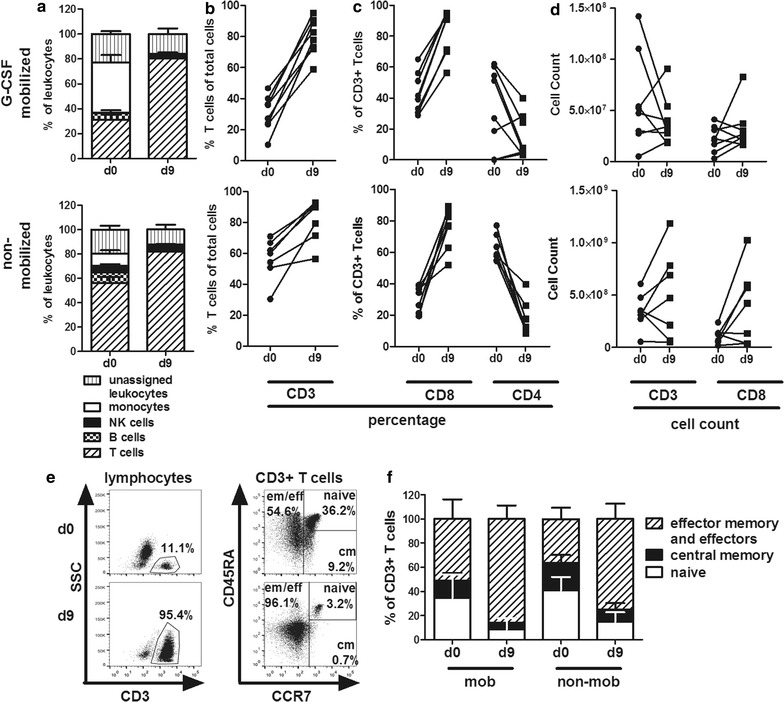
Comparison of cell composition on day 0 before peptide stimulation and day 9 at the end of expansion. **a** Relative composition of leukocytes at day 0 (d0) and day 9 (d9) after peptide stimulation from G-CSF mobilized (upper panel, n = 8) and non-mobilized donors (lower panel, n = 8). **b** Proportion of CD3+ T cells in total cells. **c** Proportion of CD8+ and CD4+ T cells within the CD3+ population. **d** Absolute counts of CD3+ T cells and of CD8+ CD3+ T cells. **e** Expression of T-cell differentiation markers on day 0 and day 9 of culture from one exemplary donor; cm, central memory; eff/em, effector and effector memory. **f** Cumulative analysis of T-cell differentiation in cultures from 7 donors of each type (G-CSF mobilized and non-mobilized donors)

For expansion of CMV- and EBV-specific T cells we utilized G-CSF mobilized (n = 8) and, for comparison, non-mobilized (n = 8) apheresis products. Our primary intention was to establish the process for G-CSF mobilized stem cell products in order to avoid a second donation and to obtain T cells prior to transplantation. For stimulation of T cells, we used two peptide pools, one for each virus, with a total of 34 peptides which represent well-characterized CD8+ and CD4+ T-cell epitopes from various proteins (latent and lytic) of CMV and EBV (Table 3). HLA restrictions of the peptides cover approximately 80% of the Central European population with at least one HLA class I match [18]. For HLA class I, we only chose peptides that elicit responses in a majority of virus carriers with the relevant HLA allotype [19–21]. Loading of PBMC with peptides was performed separately for the EBV and the CMV pool; for cultivation after loading, the two portions were joined again. As shown in Fig. 3a, apheresis products from G-CSF mobilized donors (d0) contained more than twice as many monocytes and about half as many T cells compared to non-mobilized donors. This difference disappeared after peptide stimulation and 9 days of culture (d9). Peptide stimulation increased the proportion of CD3+ total T cells and CD8+ T cells in all cultures (Fig. 3b, c). In G-CSF mobilized apheresis products, mean relative T-cell enrichment was 2.5-fold (from 31.6 ± 10.5% at day 0 to 79.2 ± 12.2% at day 9, n = 8) compared to 1.5 fold (from 54.0 ± 13.6% at day 0 to 82.1 ± 12.8%, at day 9, n = 8) in non-mobilized donors (Fig. 3a, b). As shown in Fig. 3c, T-cell enrichment was mainly due to expansion of CD8+ T cells in both groups. The percentage of CD4+ T cells was variably altered in cultures from G-CSF mobilized products, and decreased in cultures from non-mobilized donors. Absolute numbers of CD3+ total T cells and CD8+ T cells increased or decreased during cultivation (Fig. 3d). Taken together, peptide stimulation of G-CSF mobilized and non-mobilized apheresis products resulted in relative T-cell expansion, dominated by CD8+ T cells. Other cell types such as monocytes, B cells, NK cells, and other cells decreased during the culture period.

Approximately 94% of all cells collected by apheresis from G-CSF mobilized donors were lost during cryopreservation, peptide stimulation and culture until harvest of cells on day 9 (see Additional file 1: Figure S1). This loss of cells was mainly due to the depletion of granulocytes, but also of monocytes, B cells, NK cells, and T cells of supposedly irrelevant specificity.

### Reduction of the naïve compartment after expansion of virus-specific T cells

After peptide stimulation and cultivation for 9 days, composition of T-cell subsets substantially changed with respect to their status of differentiation. As shown by flow cytometric analysis in Fig. 3e exemplarily for a representative donor, the percentage of CD3+ T cells increased to 95%. Further analysis of T-cell subsets revealed an increase of effector (eff) and effector memory (em) T cells (CD45RA±, CCR7−), whereas naïve T cells (CD45RA+, CCR7+) and central memory (cm) T cells (CD45RA−, CCR7+) were reduced. Figure 3f shows the compilation of data from different G-CSF mobilized and non-mobilized donors (n = 7 each group) on day 0 before peptide stimulation and on day 9 after expansion. On average, eff/em T cells increased from 53.1 ± 16.3% to 88.8 ± 11.3% in G-CSF mobilized donors and from 36.6 ± 9.4% to 75.3 ± 12.8% in non-mobilized donors. Of note, in the day 9 products from G-CSF mobilized donors we found fewer naïve T cells (7.2 ± 9.2%) compared to products derived from non-mobilized donors (14.8 ± 7.9%). In addition, whereas the central memory T-cell compartment was reduced on average from 12.8 ± 6.5 to 4.0 ± 4.0% in G-CSF mobilized PBMC, numbers of central memory T cells from non-mobilized donors were slightly higher before and after stimulation (22.4 ± 6.7% reduced to 10.0 ± 5.6%). Thus, T-cell cultures from both mobilized and non-mobilized donors were characterized by strong expansion of CD8+ T cells with effector or effector memory phenotype.

### Specificity of the T-cell product

**Fig. 4:**
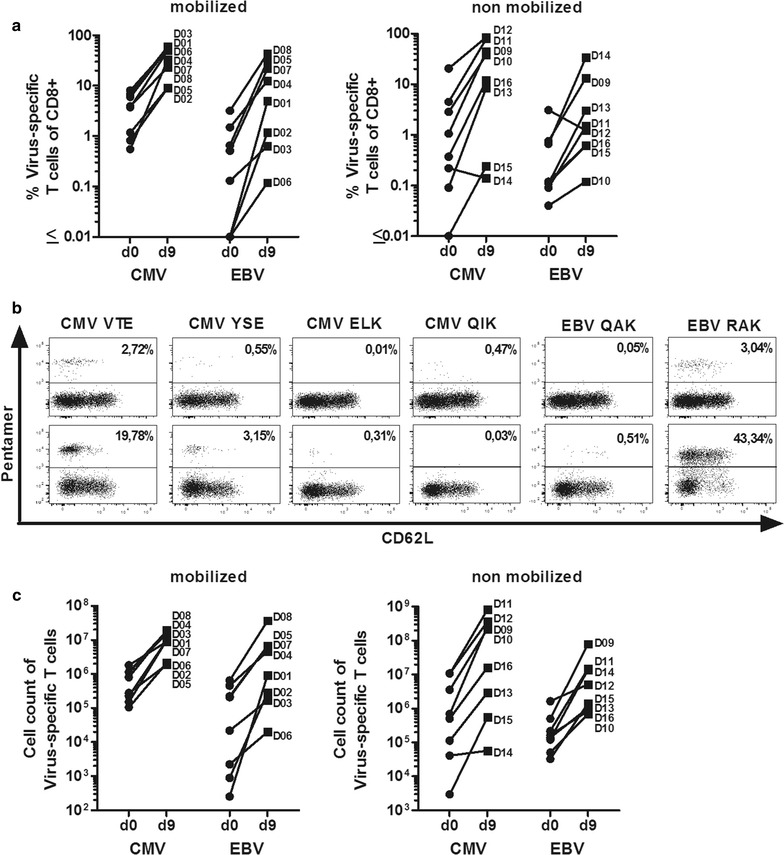
Analysis of CMV- and EBV-specific T cells before and after peptide stimulation. **a** Proportion of CMV-specific (CMV epitopes, left row) and of EBV-specific T cells (EBV epitopes, right row) on day 9 compared to day 0 as determined by HLA/peptide multimer staining (left panel: mobilized donors, n = 8; right panel: non-mobilized donors, n = 8). The cumulative proportions of T cells obtained from stainings with each applicable (HLA-compatible) multimer is shown for each donor. **b** Flow cytometric analysis for HLA/peptide multimer binding cells (% of CD8+ T cells) of one representative donor. **c** Comparision of absolute cell count of CMV-specific (CMV epitopes, left row) and EBV-specific T cells (EBV epitopes, right row) on day 9 compared to day 0 as determined by HLA/peptide multimer staining (left panel: mobilized donors, n = 8; right panel: non-mobilized donors, n = 8). **a**, **c** D01 to D12 designate the donors, as listed in Table 2

Expansion of virus-specific T cells after 9 days was determined by flow cytometry using HLA-peptide multimers appropriate for each donor’s HLA class I alleles and according to the peptide pool used for stimulation (Tables 2, 3). At the time of establishment of the protocol, not all HLA-peptide multimers were available. Table 2 shows which pentamers were used for analysis of virus-specific T cells for each donor. As shown in Fig. 4a, an increase in the relative number (cumulative percentage of multimer-binding cells) of virus-specific T cells could be observed in both G-CSF mobilized and non-mobilized donors (expansion of proportion varying from 0.6- to 94-fold for CMV and 0.4- to 496-fold for EBV, n = 16). Overall, both CMV-specific and EBV-specific CD8+ T cells were enriched in 15 of 16 cultures. Figure 4b illustrates the enrichment of antigen-specific CD8+ T cells of one representative G-CSF mobilized donor as measured by HLA-peptide multimer staining; the peptide-stimulated culture from this donor contained 20% of T cells specific for the VTE epitope from CMV pp50, and 43% of T cells specific for the RAK epitope of EBV BZLF1. Absolute expansion of virus-specific T cells was observed for each virus in each culture (Fig. 4c).

**Table 2:** HLA alleles of mobilized and non-mobilized donors

	Donor	HLA A	HLA B	HLA C	CMV multimers analyzed	EBV multimers analyzed
Mobilized	*1*	A*02:01 A*23:01	B*41:02 B*51:01	C*14:02 C*17:03	A2/NLV, A2/VLE	A2/CLG, A2/GLC, A2/YVL
	*2*	A*02:01 A*03:01	B*18:01 B*27:01	C*02:02 C*12:03	A2/NLV, A2/VLE	A2/CLG, A2/GLC, A2/YVL
	*3*	A*02:01 A*32:01	B*13:02 B*40:02	C*02:02 C*06:02	A2/NLV, A2/VLE	A2/CLG, A2/GLC, A2/YVL
	*4*	A*02:01	B*15:02 B*44:02	C*03:03 C*05:01	A2/NLV, A2/VLE	A2/CLG, A2/GLC, A2/YVL
	*5*	A*01:01	B*35:01 B*49:01	C*02:02 C*12:02	A1/YSE, A1/VTE, B35/IPS	B35/HPV, B35/EPL, B35/YPL
	*6*	A*01:01 A*03:01	B*07:02	C*07:02	A1/YSE, A1/VTE, B7/RPH, B7/TPR	B7/RPP
	*7*	A*02 A*24	B*13	C*06	A2/NLV, A2/VLE	A2/CLG, A2/GLC, A2/YVL
	*8*	A*01:01 A*24:02	B*08:01 B35*02	C*04:01 C*07:01	A1/YSE, A1/VTE, B8/ELK, B8/QIK	B8/RAK, B8/QAK
Non-mobilized	*9*	A*02	B*07 B*35	C*07	A2/NLV, A2/VLE, B7/RPH, B7/TPR, B35/IPS, C7/CRV	A2/CLG, A2/GLC, A2/YVL, A2/FLY, B7/RPP, B35/HPV, B35/EPL, B35/YPL
	*10*	A*01 A*02	B*07 B*08	nd	A1/YSE, A1/VTE, A2/NLV, A2/VLE, B7/RPH, B7/TPR, B8/ELK, B8/QIK	A2/CLG, A2/GLC, A2/YVL, A2/FLY, B7/RPP, B8/RAK, B8/QAK
	*11*	A*02 A*03	B*27 B*62	nd	A2/NLV, A2/VLE,	A2/CLG, A2/GLC, A2/YVL, A2/FLY
	*12*	A*02 A*03	B*15 B*35	nd	A2/NLV, A2/VLE, B35/IPS	A2/CLG, A2/GLC, A2/YVL, A2/FLY, B35/HPV, B35/EPL, B35/YPL
	*13*	A*02 A*24	B*44 B*51	nd	A2/NLV, A2/VLE,	A2/CLG, A2/GLC, A2/YVL, A2/FLY
	*14*	A*02 A*30	B*18 B*39	C*07	A2/NLV, A2/VLE,	A2/CLG, A2/GLC, A2/YVL
	*15*	A*02 A*26	B*07	C*06 C*07	A2/NLV, A2/VLE,	A2/CLG, A2/GLC, A2/YVL
	*16*	A*02	B*07 B*08	C*07	A2/NLV, A2/VLE,	A2/CLG, A2/GLC, A2/YVL

Peptide-pool compatible HLA alleles are listed in italic. Multimers which were available at the time and therefore used for analysis are listed for each donor in the two last columns

**Table 3:** CMV- and EBV- derived peptides used for stimulation of T cells

	Abbr.	Full sequence	-mer	Protein	HLA restriction	References
CMV no.						
1	VTE	VTEHDTLLY	9	pp50	A*01:01	Elkington et al. [41]
2	YSE	YSEHPTFTSQY	11	pp65	A*01:01	Longmate et al. [42]
3	NLV	NLVPMVATV	9	pp65	A*02:01	Diamond et al. [43]
4	VLE	VLEETSVML	9	IE-1	A*02:01	Khan et al. [44]
5	TPR	TPRVTGGGAM	10	pp65	B*07:02	Weekes et al. [45]
6	RPH	RPHERNGFTVL	11	pp65	B*07:02	Weekes et al. [45]
7	ELK	ELKRKMMYM	9	IE-1	B*08:01	Elkington et al. [41]
8	QIK	QIKVRVDMV	9	IE-1	B*08:01	Elkington et al. [41]
9	IPS	IPSINVHHY	9	pp65	B*35:01	Gavin et al. [46]
10	CRV	CRVLCCYVL	9	IE-1	C*07:02	Ameres et al. [21]
11	KYQE	KYQEFFWDANDIYRI	15	pp65	DR1, DR3	Wiesner et al. [47]
12	EHPT	EHPTFTSQYRIQGKL	15	pp65	DR11	Kern et al. [48]
13	AGIL	AGILARNLVPMVATV	15	pp65	DRB3, DRB5	Wiesner et al. [47]
14	MSIY	MSIYVYALPLKMLNI	15	pp65	DR15	Wiesner et al. [47]
15	EFFT	EFFTKNSAFPKTT	13	IE-1	DRB5*01:01	Ameres et al. [49]
16	KVYL	KVYLESFCEDVPSGK	15	pp65	DR11	Moosmann et al. unpublished
17	SVMK	SVMKRRIEEICMKVF	15	IE-1	DP3, 20, 14	Ameres et al. [49]
EBV no.						
1	CLG	CLGGLLTMV	9	LMP2	A*02:01	Lee et al. [50]
2	GLC	GLCTLVAML	9	BMLF1	A*02:01	Steven et al. [51]
3	YVL	YVLDHLIVV	9	BRLF1	A*02:01	Saulquin et al. [52]
4	FLY	FLYALALLL	9	LMP2	A*02:01	Meij et al. [53]
5	RPP	RPPIFIRRL	9	EBNA3A	B*07:02	Hill et al. [54]
6	QAK	QAKWRLQTL	9	EBNA3A	B*08:01	Burrows et al. [55]
7	RAK	RAKFKQLL	8	BZLF1	B*08:01	Bogedain et al. [56]
8	YPL	YPLHEQHGM	9	EBNA3A	B*35:01	Burrows et al. [55]
9	HPV	HPVGEADYFEY	11	EBNA1	B*35:01	Rickinson et al. [57]
10	EPL	EPLPQGQLTAY	11	BZLF1	B*35:01	Saulquin et al. [52]
11	PYYV	PYYVVDLSVRGM	12	BHRF1	DR4	Landais et al. [58]
12	VVRM	VVRMFMRERQLPQS	14	EBNA3C	DR11	Leen et al. [59]
13	FGQL	FGQLTPHTKAVYQPR	15	BLLF1	DR13	Adhikary et al. [60]
14	IPQC	IPQCRLTPLSRLPFG	15	EBNA1	DR13	Mautner et al. [61]
15	TDAW	TDAWRFAMNYPRNPT	15	BNRF1	DR15	Milosevic et al. [62]
16	VSDY	VSDYGYNEALAV	12	BNRF1	DRB3*02:02	Mautner et al. unpublished
17	AIQY	AIQYVRFLETA	11	BcLF1	DPB1*04:01	Mautner et al. unpublished

### CMV/EBV peptide-specific activation and cytokine secretion

**Fig. 5:**
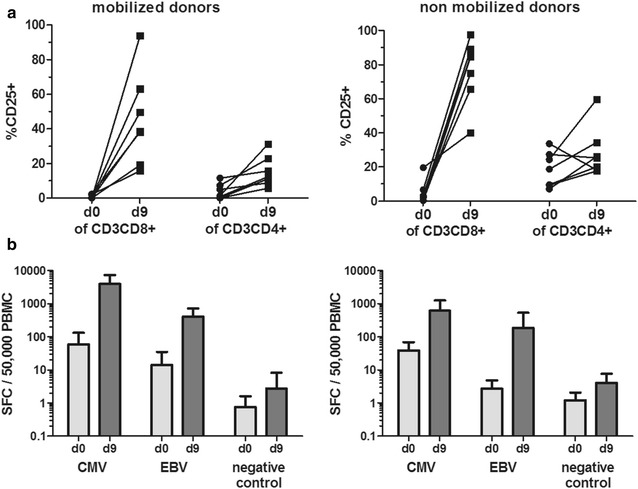
Activation and cytokine production of CMV- and EBV-specific T cells. **a** CD25 expression as activation marker is shown on day 0 and day 9 for CD4+ and CD8+ T cells of G-CSF mobilized (left panel) and non-mobilized (right panel) donors, analyzed by flow cytometry. **b** Secretion of the effector cytokine IFN-γ was analyzed by ELISpot assay after restimulation with CMV or EBV peptide pool. Shown is the number of spot-forming cells (SFC)/50,000 PBMC as mean ± SD from 5 independent donors. As a negative control, the irrelevant gp100 peptide ITDQVPFSV was used

Activation of T cells was determined by flow cytometric analysis of the expression of the IL-2Rα chain (CD25). As shown in Fig. 5a, peptide stimulation resulted in activation and significant upregulation of CD25 expression on CD8+ T cells from G-CSF mobilized (n = 7, p = 0.0021) and non-mobilized donors (n = 7, p = 0.0006), which is in line with results showing T-cell expansion in Fig. 3c. Activation was consistent but less intense (up to 31.3%) on CD4+ T cells from G-CSF mobilized (p = 0.0105), but more variable on CD4+ T cells from non-mobilized donors (p = 0.2593). Of note, we observed a significantly higher CD25 expression on non-mobilized CD4+ T cells on day 0 as compared to CD4+ T cells of G-CSF mobilized donors (p = 0.0105). Restimulation of expanded T cells from day 9 with the same peptide pools used for initial stimulation resulted in specific secretion of IFN-γ as determined by ELISpot assay (Fig. 5b). This analysis showed that the proportions of CMV- and EBV-specific, cytokine-secreting T cells were, on average, elevated between one and two orders of magnitude during peptide-driven expansion, both in mobilized and non-mobilized donors. Of note, the cumulative proportion of spot-forming CMV- and EBV-specific T cells was, on average, stronger in mobilized (mean: 4380 SFC/50.000 PBMC) than in non-mobilized donors (mean: 801 SFC/50.000 PBMC).

### Cost of manufacturing

The overall cost of the therapeutic T-cell product is approximately €11,400.- per manufacturing process. Materials costs account for 30% of the overall cost (approximately €3400.-) and include peptides and cytokines used for stimulation. Critical components are listed in Table 4. Approximately 40% of the total costs are caused by quality assessment. This includes sterility testing of the final product and during manufacturing, testing for mycoplasma and endotoxin, as well as sterility testing of critical materials such as peptide batches. The microbiological testing is outsourced to a subcontracted laboratory. Quality control costs also include cytogenetic analysis of the product as recommended by the German authorities (Paul-Ehrlich-Institute, Langen). Other costs (20%, €2100.-) include certified shipping, microbial monitoring throughout the manufacturing process, and cleaning of the GMP clean room facilities. Costs for staff (qualified person, head of quality control, head of production, technicians) were calculated on an hourly basis and amount to 10% of the overall costs.

**Table 4:** Critical materials

Material	Identification	Purpose
CMV and EBV peptide mix	Customized, jpt peptide technologies see Table 3	For stimulation
CellGro DC medium (GMP-grade medium)	Product no. 20901-0500, CellGenix	For cultivation
Interleukin-2 (Proleukin S)	PZN 2238131, Novartis	
Autologous serum	Donor-specific	
GlutaMAX™-I CTS™	Product no. A1286001, Gibco	
GMP cell differentiation bags	Product no. 170-076-402, Miltenyi Biotec	
DMSO	Pharmacy of University Hospital of Erlangen	For cryopreservation
Human serum albumin	PZN 00504775, CSL-Behring	

## Discussion

In this study we demonstrate that CMV- and EBV-specific T cells can be successfully enriched from CMV- and EBV-seropositive G-CSF mobilized donors by peptide stimulation in a GMP-compliant process, and specific T cells can be quickly obtained to allow for preventive application in patients after aSCT. Donor lymphocytes are stimulated with pools of defined viral peptides that represent dominant T-cell epitopes from various viral antigens with a range of HLA restrictions. A small portion of the G-CSF mobilized stem cell graft is sufficient as a source of T cells, eliminating the need for a second apheresis of the donor. Peptide stimulation resulted in expansion of T cells and increase of specific T cells, while reducing the proportion of naive T cells.

Several strategies to generate or isolate CMV- and/or EBV- specific T cells for adoptive transfer are currently available. Direct isolation of specific T cells without any period of cultivation generally requires the use of recombinant HLA/peptide multimers and magnetic or flow-cytometric sorting [22, 23]. This method is elegant and allows for high cell purity, but its routine clinical application is challenging since it cannot be used for all HLAs and epitopes, and a continuous stock of various manufacturing-grade multimer reagents and a separation device are needed. Antigen stimulation and subsequent isolation based on T-cell reactivity, e.g. IFN-γ secretion or CD137 up-regulation [24–28] is more versatile in terms of the T-cell specificities that can be included and can be performed within one or 2 days, but T-cell yield may be low and thus characterization of the product is often limited. Therefore, simplified approaches that take advantage of antigen-specific expansion of T cells in vitro by peptide stimulation have again attracted interest and have been explored in the clinical setting with promising results [29, 30]. One protocol [31] uses a general procedure similar to ours, but employs overlapping peptide pools covering 12 antigens from five viruses including EBV and CMV, employing approximately 2000 peptides in total, which is certainly a strong advance toward optimizing the breadth of the achievable response. In designing the present protocol, in which we used a total of 34 well-characterized peptides, 10 HLA class-I-restricted and 7 class-II-restricted peptides from each of the two viruses, we have considered it advantageous to strike a middle ground between simplicity and complexity, based on the following considerations. Our exclusive use of defined peptides with known HLA class I and II restrictions will make it possible to use HLA class I multimers (and potentially class II multimers) for a comprehensive quantitative analysis of the specific T cells in the product, and to track them in patients. Since we avoid the use of thousands of potentially irrelevant peptides, we decrease the probability that T cells with unwanted specificities that accidentally cross-react with some of these peptides will be activated and expanded. Moreover, it is very likely that our specificities of choice will be more efficiently activated if the number of potentially competing peptides in the loading reaction is limited. Still, it is important to include peptides from a range of antigens from different phases of viral replication. For CMV, it is far from clear which T-cell antigens provide the best protection from viral reactivation [28, 32, 33], and the situation for EBV is complex as well. Our choice of a limited number of epitopes and HLA restrictions will nevertheless ensure that a majority of donor/patient pairs can be covered. The idea that the use of a full-length viral antigen (or antigen-covering peptide library) is HLA-independent may be illusory, since a given viral protein will contain only epitopes presented by a finite, sometimes rather limited, number of HLA allotypes.

The presence of some remaining naïve T cells of unknown specificity in our T-cell cultures may be a safety issue in patients after aSCT, since alloreactive T cells are mainly found in the naïve compartment [34]. However, adoptive transfer of peptide-stimulated virus-specific T cells with isolation [25, 28] or without isolation [29, 30] was proven safe so far. Immunodominant peptides with known allogeneic cross-reactivity such as the EBV-derived FLR [35] were deliberately excluded from the peptide mix to minimize the risk of acute GvHD after adoptive transfer.

Furthermore, we anticipate using our T-cell product for prevention of viral reactivation at an early stage after transplantation, when most patients are still receiving calcineurin inhibitors such as cyclosporine A for acute GvHD prophylaxis. There is evidence that activated T cells become cyclosporine A resistant and therefore can exert their function in vivo despite ongoing immunosuppression [36].

A recent study described that the functionality of antiviral T cells from G-CSF mobilized stem cell grafts or after G-CSF treatment in vitro is reduced [37]. This is consistent with our observation of reduced cytokine secretion in early stages of the T-cell culture (data not shown). Furthermore, Rutella et al. described that serum of G-CSF treated healthy donors reduced lymphocyte proliferation but this effect was overcome by addition of IL-2 [38]. However, after completion of our stimulation protocol, our data show no evidence for impairment of virus-specific T cells due to the use of material from G-CSF mobilized donors. Nawa et al. [39] described a reduced stimulatory potential of monocytes from G-CSF mobilized donors in mixed lymphocyte reactions, potentially indicating reduced potential of such monocytes to stimulate T-cell responses; however, they described that T-cell function was not directly altered by G-CSF mobilization. Others described that the cell count of dendritic cells per µl PB is increased after mobilization [15, 16] bearing the advantage that these dendritic cells as APCs may contribute to effective expansion of antigen-specific T cells in protocols such as ours. During our cultivation process, however, G-CSF is absent, and we observed that 9-day cultures from G-CSF mobilized donors resulted in equal relative expansion of virus-specific T cells in comparison to cultures from non-mobilized donors, and T-cell function, as measured in ELISpot, was even better for mobilized donors. In line with our results, Samuel et al. showed that a successful expansion of CMV-reactive T cells from G-CSF mobilized donors is possible [40]. In addition, Chevallier et al. reported no significant differences in the composition of T-cell subsets of peripheral blood of G-CSF mobilized and non-mobilized donors [16]. The reduction of naïve T cells in the products of both donor types is encouraging, as the naive T-cell compartment is considered the main source of alloreactive T cells [34].

## Conclusions

One major advantage of this manufacturing protocol is its cost efficiency. First, in comparison to current standard therapies of CMV or EBV reactivation (ganciclovir, rituximab, etc.), specific T-cell therapy in general is a competitive option in terms of costs. Second, among possible protocols to prepare specific T cells, the present one is particularly economic, since it does not depend on expensive proprietary technology for specific cell cultivation or isolation, but uses only general-purpose machinery that has multiple uses in transfusion medicine departments. Multiple doses can be produced in one manufacturing run, and can be stored for repeated application if necessary. Our protocol is versatile, and will allow for including peptides from additional viral or other microbial pathogens without any changes in the workflow.

We are currently conducting a phase I/IIa clinical trial exploring a preventive strategy using this T-cell product in patients after aSCT (Eudract 2012-004240-30). Peptide-stimulated T cells are stored over liquid nitrogen and administered at a dose of 5 × 10^4^/kg CD3+ cells/kg body weight on days 30, 60, and 90 after aSCT. Aside from safety we aim at demonstrating efficacy of this T-cell product. In a more long-term perspective, we are confident that simplified protocols to prepare CMV/EBV-specific T-cells for adoptive therapy after aSCT will help make this therapy more widely available, especially for prophylactic intervention to prevent reactivation and disease caused by these widespread viruses. We believe this may decrease virus reactivation rates, thereby reducing hospitalization time and need for medication prone to side effects, and improving quality of life for patients after aSCT.

## Additional file

**Additional file 1: Figure S1.** Yield of cells during manufacturing process. Proportion of cell count on d-1, d0, and d9 of manufacturing process compared to cell count of starting material before cryopreservation of 5 independent manufacturing processes of material of mobilized donors.

## Abbreviations

APC: antigen-presenting cell
aSCT: allogeneic stem cell transplantation
cm: central memory
CMV: cytomegalovirus
DLI: donor lymphocyte infusion
DMSO: dimethyl sulfoxide
EBV: Epstein–Barr virus
G-CSF: granulocyte colony-stimulating factor
GMP: good manufacturing practice
GvHD: graft-versus-host disease
HLA: human leukocyte antigen
HSA: human serum albumin
IFN: interferon
IL: interleukin
NK: natural killer
PB: peripheral blood
eff: effector
em: effector memory
PBMC: peripheral blood mononuclear cells
PBS: phosphate-buffered saline
SFC: spot-forming cells
