# Supplementary figures and images for: Clinical-grade generation of peptide-stimulated CMV/EBV-specific T cells from G-CSF mobilized stem cell grafts
Source: J Transl Med. 2018 May 9;16:124. doi: 10.1186/s12967-018-1498-3 (PMC5941463; doi:10.1186/s12967-018-1498-3)

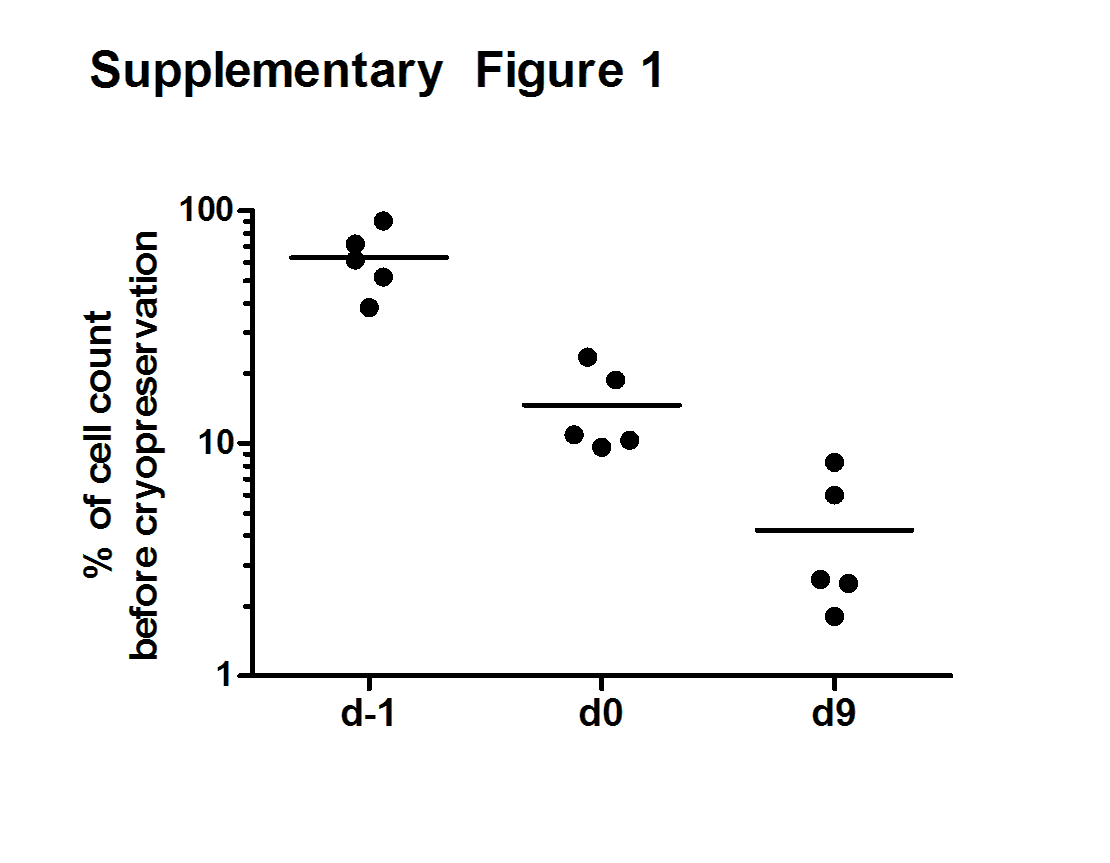

Supplement: Supplementary file 1 — Additional file 1: Figure S1. Yield of cells during manufacturing process. Proportion of cell count on d-1, d0, and d9 of manufacturing process compared to cell count of starting material before cryopreservation of 5 independent manufacturing processes of material of mobilized donors. [file 12967_2018_1498_MOESM1_ESM.tif]
